# Rethinking Human Embryo Research Policies

**DOI:** 10.1002/hast.1215

**Published:** 2021-02-25

**Authors:** Kirstin R. W. Matthews, Ana S. Iltis, Nuria Gallego Marquez, Daniel S. Wagner, Jason Scott Robert, Inmaculada de Melo‐Martín, Marieke Bigg, Sarah Franklin, Soren Holm, Ingrid Metzler, Matteo A. Molè, Jochen Taupitz, Giuseppe Testa, Jeremy Sugarman

**Keywords:** embryo, fourteen‐day limit, research ethics, guidelines, policy

## Abstract

*It now seems technically feasible to culture human embryos beyond the “fourteen‐day limit,” which has the potential to increase scientific understanding of human development and perhaps improve infertility treatments. The fourteen‐day limit was adopted as a compromise but subsequently has been considered an ethical line. Does it remain relevant in light of technological advances permitting embryo maturation beyond it? Should it be changed and, if so, how and why? What justifications would be necessary to expand the limit, particularly given that doing so would violate some people's moral commitments regarding human embryos? Robust stakeholder engagement preceded adoption of the fourteen‐day limit and should arguably be part of efforts to reassess it. Such engagement could also consider the need for enhanced oversight of human embryo research. In the meantime, developing and implementing reliable oversight systems should help foster high‐quality research and public confidence in it*.

## Article

New technology makes it feasible to culture human embryos several days longer than before, beyond the fourteen‐day limit on such research. A deliberative process with broad stakeholder participation and public outreach is essential in reassessing that carefully established limit.

In 2016, two independent teams, one in the United States and one in the United Kingdom, reported culturing human embryos in vitro up to fourteen days after fertilization.^1^ The experiments were terminated by day fourteen or at the formation of the primitive streak to respect an international norm and, in the case of the U.K. team, to be in compliance with the law. This norm is known as the *fourteen‐day rule* or *fourteen‐day limit*.

The fourteen‐day limit for embryo research was proposed first in a 1979 U.S. Department of Health, Education and Welfare (DHEW) report and later in the 1984 U.K. *Report of the Committee of Inquiry into Human Fertilisation and Embryology*, commonly known as the Warnock Report.^2^ The preparation of both documents involved extensive public and stakeholder engagement, including in‐person meetings and the review of hundreds of letters from the public. The reports focused on the clinical practice of in vitro fertilization (IVF) and its related research.

Fourteen days was not chosen arbitrarily. Rather, specific reasons were offered for adopting it within these reports, and additional justifications were articulated in the three decades following their release.^3^ First, the primitive streak appears around fourteen days, marking the first visible sign of significant organization of the embryo just prior to neural tube formation, and it is an event that can be easily identified in culture. Furthermore, twinning does not seem to occur after fourteen days,^4^ suggesting that this is the point of individuation, leading some to claim that destruction of embryos prior to this point does not destroy individuals.^5^

The diverse international regulations on human embryo research adopted since the limit was proposed reflect different normative views. Some countries (including Austria, Germany, Italy, Russia, and Turkey) ban human embryo research.^6^ The United Kingdom includes the fourteen‐day limit in the Human Fertilisation and Embryology Act of 1990, which permits human embryo research after approval by a regulatory authority. Other countries (including Canada, South Korea, and Sweden) allow certain human embryo research but have enshrined the fourteen‐day limit into law. Still other countries (Israel among them) regulate human embryo research but do not specify a particular limit. In the United States, researchers cannot use federal funds for human embryo research, but privately funded research beyond fourteen days is possible. Nevertheless, to date, scientists appear to respect the fourteen‐day limit, regardless of its legal status in their country.

The fourteen‐day limit is often framed as an ethical compromise to permit human embryo research despite the moral objections of some. However, when the limit was established, it was not technically possible to culture human embryos beyond five or six days. Thus, the fourteen‐day limit imposed no tangible restriction to human embryo research, and proponents of this research lost little in agreeing to it. A policy that prohibits what is not scientifically feasible does not impinge on practice. In contrast, those who opposed all human embryo research relinquished their preference for a complete prohibition. Only now that technology has advanced does the fourteen‐day limit appear to reflect a compromise between some human embryo research proponents and opponents.

## Arguments for and against Changing the Fourteen‐Day Limit

Following the 2016 publications describing the technological feasibility of culturing human embryos beyond the fourteen‐day limit, scientists and others began discussing its relevance.^7^ Those pushing to abandon the limit see it as a technical constraint, rejecting the view that research beyond this point is unethical.^8^ Most arguments for moving the limit highlight the knowledge obtainable beyond day fourteen. After all, there is, in fact, limited knowledge of early human development, with theories about embryo development based in large part on animal studies and fixed slides of embryos. Arguments in favor of moving the limit further contend that research beyond day fourteen could help improve IVF results and expand understanding of the effect of toxic chemicals and environmental factors on embryos, the causes of infertility, and early developmental issues such as neural tube closure.^9^

In addition, some parties have argued that the fourteen‐day limit is now obsolete because it is unclear whether and how it applies to human embryoid research. Embryoids are derived from pluripotent stem cells and seem to mimic early human development—that is, they recapitulate, in vitro, salient aspects of the early stages of embryo development—although they are not embryos.^10^ Some see them as alternatives to using human embryos in research.^11^ However, embryoids do not progress linearly; instead, they mimic specific developmental points. For example, an embryoid could mimic gastrulation (around day seventeen in actual human embryos) in less than fourteen days but without having developed the primitive streak. Furthermore, embryoids approximate only the early embryo; they cannot become fully functional human embryos. Therefore, many scientists suggest that research on embryoids should not be guided by the fourteen‐day limit nor regulated as human embryo research. As a related matter, it is unclear whether many national human embryo research laws and guidelines pertain to embryoid research.^12^

At the same time, others offer reasons to maintain the fourteen‐day limit, at least for now. Those who object to all research that destroys human embryos would likely not support any expansion of research. For those who accept some human embryo research but have moral reservations about certain forms of it, the limit serves as an ethical limit. Indeed, on such grounds, Mary Warnock, Magdalena Zernicka‐Goetz (the leader of the United Kingdom‐based lab that cultured human embryos up to day fourteen in 2016), and others opine that it should not be changed without more rigorous discussion and debate.^13^ Others argue that the fourteen‐day limit was a compromise and should stay in place despite new technical possibilities and research opportunities. In their view, abandoning the limit, now that it is only policy and not technology standing in the way of research, would suggest that scientists cannot be trusted to respect policies developed through compromise and could undermine public trust in science. It has also been suggested that valuable research could be conducted prior to day fourteen to help in understanding early pregnancy loss, improve IVF, and investigate environmental factors on the embryos. Since research on embryos approaching fourteen days has only recently become possible, much can still be learned from additional work prior to day fourteen without changing or abandoning the limit. Further, the results of such research could inform future public and stakeholder engagement on the question whether research beyond fourteen days should be permitted.

## Challenges with Developing New Policies

Among those calling to change the fourteen‐day limit, there is no agreement about what, if anything, should replace it or what would justify adopting different limits or criteria for research on older embryos. Based on this lack of consensus and the global diversity of current human embryo and embryonic stem cell research policies, it is reasonable to expect that various policies will be developed to fit the values and preferences of different jurisdictions. While understandable, this diverse set of policies might complicate scientific work and limit scientists’ ability to collaborate, present, and publish internationally.

There are several possible options for replacing the fourteen‐day limit. One possibility is to identify a new date, such as twenty‐eight days, or a biological event, such as neural tube closure, which begins around seventeen days. If a biological event is used, it must be easily identifiable so that researchers can observe it without perturbing their experiments and do not accidently exceed the limit. The fourteen‐day limit meets this criterion for embryos (but not embryoids) because it is linked to the visible formation of the primitive streak, allowing multiple noninvasive checks to ensure adherence.^14^ In fact, some national laws use the date, some use the biological event, and others use both. However, there is no consensus on what a new time point or event should be.

Another option is to have no established limit but to review human embryo research intended to exceed fourteen days on a case‐by‐case basis. Using this approach, each proposed research project would be evaluated in light of a set of criteria, similar to how research ethics committees such as institutional review boards oversee human research or how stem cell research oversight (SCRO) committees review research involving some types of human pluripotent stem cells. The review process would require investigators to articulate why the expected knowledge justifies culturing embryos beyond fourteen days. This option would, however, necessitate creating explicit guidelines, establishing oversight bodies with sufficient expertise and authority, and implementing transparent procedures to promote public confidence in the decisions rendered. Although such a system might be subject to many of the criticisms of contemporary human research oversight processes, including variability in decisions made by different review boards, a formal oversight process that has clearly defined procedures is, as we argue below, important for human embryo research.^15^

## The Need for Transparency and Stakeholder Engagement

The robustness and longevity of the fourteen‐day limit may owe much to the deliberative and transparent process used to develop it. Accordingly, both public and stakeholder engagement seems critical to determining what, if any, policy changes are appropriate.

Given the widespread acceptance of the fourteen‐day limit, contested value judgments about embryos, the concerns about extending the limit, and the uncertain benefits from extending it,^16^ a deliberative process that entails broad stakeholder participation and public outreach is needed for determining whether a new embryo research limit should be implemented and, if so, which one. The range of stakeholders to be engaged includes patients, embryo donors, scientists, clinicians, funders, and religious leaders. Engagement should ideally help to identify trustworthy solutions that limit offense to those who object to human embryo research. Failing to engage relevant stakeholders risks missing important perspectives on this complex issue and the loss of public trust in science. In contrast, public dialogue, though requiring significant time and resources to be done well, seems well suited to help advance science as a good worthy of public support.

It is worth recalling that, when developing the 1979 DHEW report, the committee conducted eleven hearings in nine cities across the United States and reviewed more than two thousand documents received from the public.^17^ Similarly, the U.K. process to develop the Human Fertilisation and Embryology Authority involved both the Warnock commission and its report (including minority dissents) as well as six years of public discussion before the law was passed.^18^ More recently, HFEA conducted a multiyear consultation process to develop guidelines for mitochondrial replacement therapy, including reviewing scientific and ethical issues as well as conducting open public dialogues.^19^ Engaging diverse publics to identify and understand broad concerns linked with more focused engagement with stakeholders can help develop a policy with appropriate justifications.

## Oversight of Human Embryo and Embryoid Research

Despite the ethical issues associated with embryo research, there has long been recognition that independent oversight is lacking in some jurisdictions. Whether or not the fourteen‐day limit is maintained, a system for reviewing and overseeing human embryo research in all jurisdictions where the research is permitted would be desirable. This is particularly vital in the United States, where there is no centralized authority over human embryo research (with the exclusion of research intended for clinical therapies that the U.S. Food and Drug Administration reviews). Other than that, no federal requirement for local or institutional oversight of human embryo research exists. However, the United States is not unique; other countries, including China and India, also do not have national authorities overseeing human embryo research.^20^

Appropriate oversight could be established by law, as is the case in the United Kingdom, or through professional standards. In the United Kingdom, the HFEA specifies how to obtain licenses, lists all research under licensure, and holds public consultations when considering any changes in its mandate. It uses transparent oversight processes that ensure appropriate review and enforcement of limits.^21^

In contrast, the International Society for Stem Cell Research issues guidelines that provide recommendations for human embryo research oversight that could be undertaken through institutional and professional channels. ISSCR suggests that existing institutional SCRO committees are one means of meeting these recommendations.^22^ Adding human embryo research oversight to the responsibilities of SCRO committees or convening a similar committee for this purpose acknowledges the sensitive nature of human embryo research and ensures it is vetted scientifically and ethically. It would also provide independent ethical oversight that this research might not have otherwise.

Oversight responsibility could be added to existing committees, or new ones could be formed to review human embryo and embryoid research. Regardless of the means, it will be important to specify requirements for the composition of such committees and the criteria used for reviewing research, akin to the existing requirements for institutional review boards (and, outside the United States, research ethics committees). Human embryo oversight committees should also be subject to some form of external review or accreditation to help ensure a consistent and competent process for research oversight.

## Moving Forward

Arguments for changing the fourteen‐day limit have emerged following the development of novel technical innovations and a scientific desire to further understand early human development. However, although the technological possibility of doing so and the promise of new knowledge are necessary conditions for considering a change to the fourteen‐day limit, they alone seem to be insufficient. Deliberations about changing the limit should also take into account the ethical arguments for and against human embryo research, particularly regarding research on more developed embryos. Scientists should not conduct these discussions in isolation. The fourteen‐day limit was adopted after extensive and transparent public and stakeholder engagement in the United Kingdom and the United States. Any possible change would benefit from a comparable process.^23^

Scientists should also work with interested stakeholders to develop processes for rigorous and transparent oversight of all human embryo research—even research on embryos prior to fourteen days, which is currently subject to little or no oversight in some jurisdictions. Such oversight could enhance public trust in the scientific process and ensure accountability. Oversight guidelines should include criteria for the composition and function of oversight committees and for the review and approval of human embryo research. Oversight committees should also have adequate stakeholder representation. Proper oversight acknowledges the sensitive nature of human embryo research, promotes transparency and high‐quality science, and can ultimately contribute to and enhance public trust in science.

## Acknowledgments

The authors would like to thank the Baker Institute staff including Daniel Morali and Sharon Tsao for their work on the project. Funding for the project was provided by The Greenwall Foundation “Making a Difference” grant, a Brocher Foundation workshop grant, and the Baker Institute Center for Health and Biosciences.

## Disclosures

Jeremy Sugarman and Jochen Taupitz are members of the Merck KGaA Bioethics Advisory Panel and Stem Cell Research Oversight Committee. Sugarman is a member of IQVIA's Ethics Advisory Panel and has consulted for Portola Pharmaceuticals, Inc. Matteo Molè works in a lab that conducts human embryo research.
